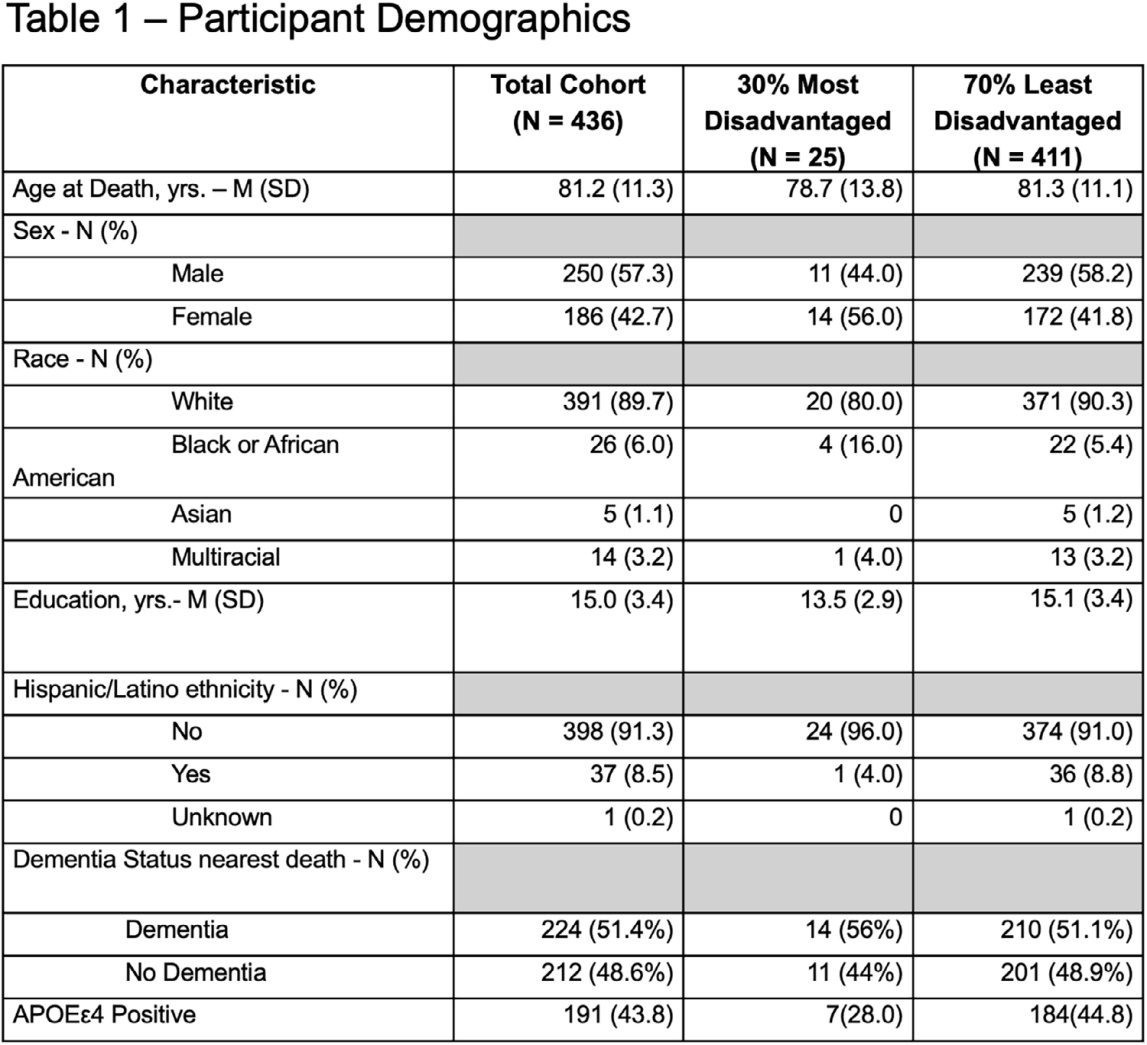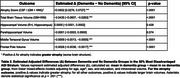# Association of Neighborhood‐Level Disadvantage with Antemortem MRI Brain Volumes in a Multi‐site Brain Bank Donor Cohort

**DOI:** 10.1002/alz70861_108929

**Published:** 2025-12-23

**Authors:** Angel Hammond, Ruvini Navaratna, Jason F Moody, Amanda DeWitt, Michael L Alosco, Alireza Atri, Kelly M. Bakulski, Thomas G Beach, James B. Brewer, Carmen Carrion, Joshua Chodosh, Anthony Fabio, Sarah Tomaszewski Farias, Victor W Henderson, Julia K. Kofler, Jessica B. Langbaum, Rebecca J Lepping, Jennifer H Lingler, Jonathan D Mahnken, Karyn Marsh, Adam P Mecca, Oanh L. Meyer, Jill K Morris, Judith A. Neugroschl, Maureen K. O'Connor, Henry L Paulson, Eric M. Reiman, Patricia Rodriguez Espinoza, Mary Sano, Andrew J. Saykin, C. Elizabeth Shaaban, David L Sultzer, Rachel A. Whitmer, Thomas Wisniewski, Carolyn W. Zhu, William R. Buckingham, W. Ryan Powell, Amy J.H. Kind, Tobey J. Betthauser, Barbara B. Bendlin

**Affiliations:** ^1^ Center for Health Disparities Research, University of Wisconsin School of Medicine and Public Health, Madison, WI USA; ^2^ Wisconsin Alzheimer's Disease Research Center, School of Medicine and Public Health, University of Wisconsin‐Madison, Madison, WI USA; ^3^ Boston University Chobanian & Avedisian School of Medicine, Boston, MA USA; ^4^ Banner Health, Saint Louis, MO USA; ^5^ Michigan Alzheimer's Disease Research Center, University of Michigan Medical School, Ann Arbor, MI USA; ^6^ Banner Health, Sun City, AZ USA; ^7^ University of California, San Diego, La Jolla, CA USA; ^8^ Yale University School of Medicine, New Haven, CT USA; ^9^ NYU Grossman School of Medicine, New York, NY USA; ^10^ University of Pittsburgh, Pittsburgh, PA USA; ^11^ University of California, Davis, Sacramento, CA USA; ^12^ Stanford University, Palo Alto, CA USA; ^13^ Banner Alzheimer's Institute, Phoenix, AZ USA; ^14^ Hoglund Biomedical Imaging Center, University of Kansas Medical Center, Kansas City, KS USA; ^15^ University of Kansas Alzheimer's Disease Research Center, Fairway, KS USA; ^16^ New York University Grossman School of Medicine, New York, NY USA; ^17^ Yale University, New Haven, CT USA; ^18^ University of California, Davis School of Medicine, Sacramento, CA USA; ^19^ University of Kansas Medical Center, Alzheimer's Disease Research Center, Fairway, KS USA; ^20^ Icahn School of Medicine at Mount Sinai, New York, NY USA; ^21^ Boston University School of Medicine, Boston, MA USA; ^22^ Geriatric Research Education and Clinical Center, VA Bedford Healthcare System, Bedford, MA USA; ^23^ University of Michigan, Ann Arbor, MI USA; ^24^ Banner Sun Health Research Institute, Sun City, AZ USA; ^25^ Stanford University School of Medicine, Palo Alto, CA USA; ^26^ Indiana Alzheimer’s Disease Research Center, Indiana University School of Medicine, Indianapolis, IN USA; ^27^ University of Pittsburgh Alzheimer's Disease Research Center (ADRC), Pittsburgh, PA USA; ^28^ University of California, Irvine, Irvine, CA USA; ^29^ School of Medicine, University of California, Irvine, Irvine, CA USA; ^30^ University of California, Davis, Davis, CA USA; ^31^ Icahn School of Medicine, Mount Sinai Hospital, New York, NY USA; ^32^ Wisconsin Alzheimer’s Disease Research Center, University of Wisconsin School of Medicine and Public Health, Madison, WI USA; ^33^ Wisconsin Alzheimer's Disease Research Center, University of Wisconsin School of Medicine and Public Health, Madison, WI USA

## Abstract

**Background:**

Higher neighborhood disadvantage, indexed by the Area Deprivation Index (ADI), is linked with lower MRI‐measured brain volume in cognitively unimpaired individuals. We investigated whether ADI is associated with longitudinal antemortem MRI‐derived brain volumes across the cognitive continuum in a multi‐site brain bank cohort.

**Method:**

We analyzed 724 T1‐weighted MRI scans from 436 brain donors (Table 1) across 14 Alzheimer’s Disease Research Centers housed in the National Alzheimer’s Coordinating Center. Images were processed using a longitudinal pipeline. Donor addresses were geocoded and linked to ADI percentiles at death year. ADI was binarized (30% most vs 70% least disadvantaged) and used to predict brain volumes in linear mixed‐effects models. Outcomes included atrophy score (CSF / [GM + WM]), total brain volume, and hippocampal, parahippocampal, middle temporal gyrus, and frontal pole volumes, all normalized to intracranial volume. Covariates included age, age², sex, education, and dementia status (CDR 0–0.5 = no dementia; 1–3 = dementia). An exploratory ADI × dementia interaction was tested. Estimated marginal means and contrasts were extracted to interpret ADI effects within each dementia stratum. Random intercepts for participant and site were included.

**Result:**

A significant ADI × dementia interaction was observed for global atrophy (p = .005) and several other outcomes. Among participants in the 30% most disadvantaged group, dementia was associated with greater brain volume differences compared to no dementia (Table 2). Dementia was linked to greater global atrophy (Δ = +0.0502, 95% CI [0.0276, 0.0727]), lower total brain volume (Δ = −0.0435), middle temporal gyrus (Δ = −0.0009), and frontal pole (Δ = −0.0028), all *p* < .0001. A marginal trend was observed for parahippocampal volume (p = .074); no difference was found for hippocampus.

**Conclusion:**

Dementia was associated with greater global atrophy and lower brain volumes among disadvantaged individuals. These findings suggest that socioeconomic disadvantage may amplify dementia‐related brain changes. The small number of participants with both dementia and high disadvantage (n = 14) is a limitation, underscoring the need for greater socioeconomic representation in future studies.